# Human herpesvirus-8 (HHV-8) sero-detection and HIV association in Kaposi's sarcoma (KS), non-KS tumors and non-neoplastic conditions

**DOI:** 10.1186/1750-9378-3-10

**Published:** 2008-06-30

**Authors:** Amos R Mwakigonja, Pawan Pyakurel, Parviz Kokhaei, Fatemeh Pak, Leonard K Lema, Ephata E Kaaya, Peter Biberfeld

**Affiliations:** 1 Immunopathology Laboratory, Department of Oncology-Pathology, Cancercentrum Karolinska (CCK), Karolinska University Hospital Solna/Karolinska Institutet, Stockholm, Sweden; 2 Department of Pathology, Muhimbili University of Health and Allied Sciences (MUHAS), Dar es Salaam, Tanzania; 3 Department of Immunology, Semnan Medical University, Semnan, Iran; 4 Immune and Gene Therapy Lab., Cancercentrum Karolinska (CCK) Karolinska University Hospital Solna/Karolinska Institutet, Stockholm, Sweden; 5 Department of Surgery, MUHAS and Muhimbili National Hospital (MNH), Dar es Salaam, Tanzania

## Abstract

**Background:**

The association of the human herpesvirus-8/Kaposi's sarcoma (KS)-associated herpesvirus (HHV-8/KSHV) serology with various malignancies in Tanzania is not currently well established while previous studies were based on either PCR or immunofluorescence assays [IFA] but not with a sensitive enzyme-linked immunosorbent assay (ELISA). Selected archival diagnostic biopsies (n = 184) and sera from indigenous patients with KS (n = 120), non-KS tumors (n = 24) and non-neoplastic lesions (n = 40) at Muhimbili National Hospital (MNH), Tanzania, were evaluated by diagnostic histopathology, immunohistology [anti-HHV-8 latency-associated nuclear antigen (LANA)] and serology for HIV (ELISA) and HHV-8 (IFA and ELISA).

**Results:**

About 66.3% (n = 122) cases including AIDS-associated Kaposi's sarcoma (AKS) (n = 93), reactive conditions (n = 28) and only one non-KS tumour were HIV positive. Endemic KS (EKS) patients were mostly males (96.3%, 26/27) who were less (69.9%, 65/93) predominant in AIDS-associated (AKS). A high (89%) percentage of patients with anti-HHV-8 antibodies was found in the cohort including the HIV positive (92%) cases, males (81.2%), KS patients (93%), non-KS tumors (92%), and reactive conditions (75%). All HHV-8 seronegative KS cases were nodular stage whereas both sera and corresponding biopsies from early stage KS were HHV-8+. Assay sensitivity, positive predictive value (PPV) and specificity were 98.6%, 93.5% and 16.7% for IFA and 93.5%, 98.6% and 50.0% for ELISA respectively.

**Conclusion:**

HHV-8 seroprevalence at MNH appears high as expected among AKS cases and males but also in non-KS patients. ELISA showed a combination of high HHV-8 sensitivity as well as higher PPV and specificity than IFA which however, showed higher sensitivity. The apparent stage-dependent, inverted serum HHV-8 immunoreactivity supports a notion of viral immune-segregation during KS development. Routine HHV-8 screening should be considered particularly in patients at risk of KS and for selection of blood/organ donations.

## Background

The HIV and AIDS epidemic has dramatically increased the frequency of different malignancies particularly Kaposi's sarcoma (KS) and certain malignant lymphoma (ML) which are associated with the novel human herpesvirus type 8 (HHV-8)/Kaposi's sarcoma-associated herpes virus (KSHV) and have become a major health concern in sub-Saharan Africa including Tanzania [1-3]. The prevalence of HHV-8 varies from high-endemicity (30–70%) areas (sub-Saharan Africa), intermediate (5–20%) [Mediterranean] and low (≤ 5%) (Northern Europe, USA, south-east Asia and Japan) reflecting the KS and HIV epidemiology [4,5]. HHV-8 has a high (≈50%) prevalence in the healthy Tanzanian population (blood donors) although the prevalence in Tanzanian patients with and without tumors is poorly documented [6]. Seroconversion to HHV-8 precedes and therefore is predictive of KS development [4] and serodetection may also help to confirm KS diagnosis in suspect and/or borderline tumour lesions. HHV-8 transmission is known to occur both horizontally (orally, sexually and parenterally) and vertically (mother-to-child) particularly in endemic areas [7,8]. Obviously, large-scale HHV-8 screening would be beneficial and allow preventive/therapeutic interventions including possible anti-HHV-8 vaccination in at-risk populations, but this is not yet well documented in Africa, particularly Tanzania. Previous studies on HHV-8 in Tanzania were based on polymerase chain reaction (PCR) assay which is not readily and widely available in resource-constrained developing countries [5,6,9]. Furthermore, the tested non-KS Tanzanian sera in the previous study by Massambu et al., (2003) [6] were very few calling for the current larger study of hospital patients (KS, non-KS neoplasia and non-malignant clinical conditions), for comparison with our previous reports including that on healthy blood donors, which was based on immunofluorescence assay (IFA) and real-time PCR [5], The use of IFA necessitates culturing and subsequent processing of a HHV-8+ body cavity-based lymphoma (BCBL-1) cell line or another B-cell line (BCP-1) which may not be readily available and affordable in Tanzania particularly on a routine/large scale screening basis. Enzyme-linked immunoassays (ELISA) do not involve the use of cell cultures and thus provide an easy and more affordable screening method for HHV-8 infection. HHV-8 sero-detection technology is still a developing area and new assays like ELISAs may not yet have been tested in the high-endemicity African sera as compared to IFA [4,10]. Our present study therefore, compares IFA and ELISA for HHV-8 serology of indigenous Tanzanians thus establishing an assay for possible routine use. Furthermore, although it is known that HIV transactivates HHV-8/KSHV infection mostly by its Tat protein, the association of HHV-8 and HIV with non-KS neoplasia and non-malignant (reactive) conditions in Tanzania has not yet been examined and is therefore evaluated in the present study [6].

## Results

### Demography

**Figure 1:**
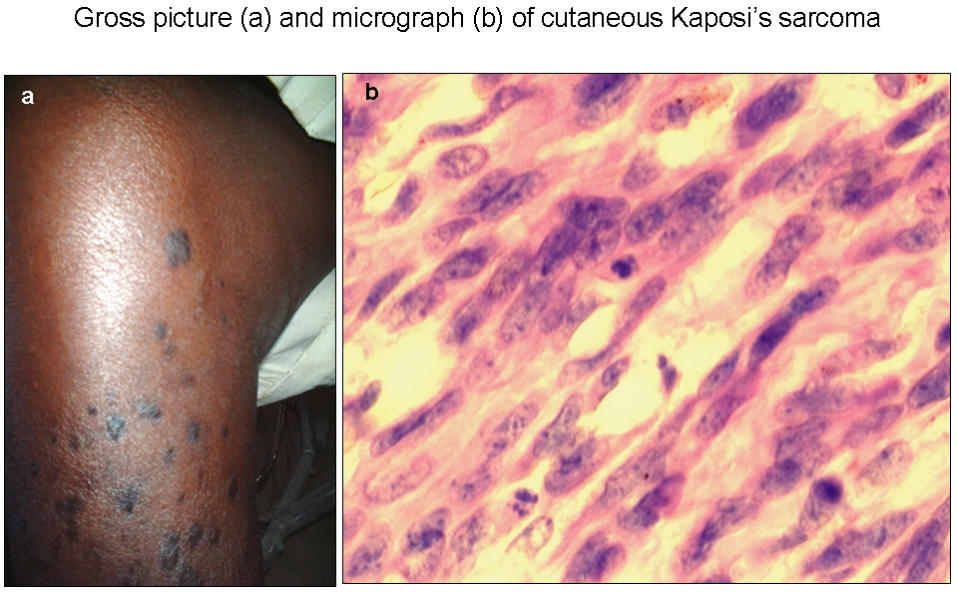
Cutaneous nodular AKS: gross picture of the right leg of an African patient showing multiple nodules and macular (patch/plaque) lesions [a] and a micrograph showing predominance of spindle cells (SC) in a H & E section [b] × 400.

A total of 184 selected biopsies and corresponding sera available at MNH/MUHAS between 1990 and 2001 from indigenous African patients were included in the study. Of these patients, 120(65%) had KS, 24(13%) non-KS tumors and 40(22%) non-malignant (reactive) conditions. The male:female ratio was 2.75:1 for KS, 3:1 for non-KS tumors and almost 1:1 for reactive, non-malignant conditions [Figure 1(a)] and (Table 1). The mean age for males was 36.6 years ranging from 12–70 and for females 32.7 years ranging from 17–50 [Table 1].

**Table 1:** Demographic characteristics of studied MNH patients with KS, non-KS tumors and reactive lesions in association with HIV serostatus

**Histological diagnosis**	**Age characteristics (years)**	**Age-group (years)**		**Sex**		**HIV serostatus**		**Overall cohort(Years)**
					
		**Adults (≥15)**	**Children (<15)**	**Male**	**Female**	**Positive**	**Negative**	
**Kaposi's sarcoma (KS) [120 cases]**	Mean age	35.8	na*	36.6	32.7	35.5	37.2	**35.6**
	Age-Range	17–70	na*	12–70	17–65	12–70	19–65	**12–70**
**Total (%)**		**119 (99.2)**	**1 (0.8)**	**88 (73.3)**	**32 (26.7)**	**93 (77.5)**	**27 (22.5)**	**120 (100.0)**
**Non-KS malignancies [24 cases]**	Mean age	36.5	8.5	31.1	22.4	na *	29.6	**29.2**
	Age-Range	15–63	5–14	5–63	7–27	na *	5–63	**5–63**
**Total (%)**		**18 (75.0)**	**6 (25.0)**	**18 (75.0)**	**6 (25.0)**	**1 (4.2)**	**23 (95.8)**	**24 (100.0)**
**Non-malignant cases [40 cases]**	Mean age	34.9	8.0	37.5	26.1	31.8	31.2	**31.6**
	Age-Range	18–52	2–11	9–52	2–47	2–52	9–44	**2–52**
**Total (%)**		**36 (90.0)**	**4 (10.0)**	**21 (52.5)**	**19 (47.59**	**28 (70.0)**	**12 (30.0)**	**40 (100.0)**
**Grand total (%)**		**173 (94.0)**	**11 (6.0)**	**127 (69.0)**	**57 (31.0)**	**122 (66.3)**	**62 (33.7)**	**184 (100.0)**

Key: na = not applicable, * = only one case available.

### Histopathology

Of the KS cases, most (68.7%, 82/120) were nodular stage [Figure 1(b)] with cutaneous (87.8%, 72/82) or lymphadenopathic (12.2%, 10/82) localization [Table 2(a)]. The non-KS tumors included malignant lymphomas (45.8%), epithelial cancers (25.0%), soft tissue tumors (25.0%) and one neuroendocrine tumor (4.2%) [Table 2(b)]. Most of non-neoplastic biopsies came from patients with reactive lymphadenitis and other inflammatory conditions.

**Table 2:** Histological diagnosis of (a) KS patients and (b) non-KS malignancies

**(a) KS cases**	**Number**	**Percentage**
Lymphadenopathic nodular KS	10	8.7
Nodular KS	72	60
Patch KS	21	17.4
Plaque KS	17	13.9

**Total**	**120**	**100**

**(b) Non-KS malignancies**	**Number**	**Percentage**
Lymphomas	11	45.8
Epithelial tumors	6	25
Neuroendocrine tumors	1	4.2
Soft tissue tumors	6	25

**Total**	**24**	**100.0**

### HIV serology

Most (66.3%, 122/184) studied sera were HIV positive (Table 1). Females were only 3.7% (1/27) among EKS patients and 30.1% (28/93) in AKS whereas males were predominant in both KS types which differences were statistically significant (χ^2 ^= 7.96, p = 0.0048) [Table 1].

Most KS cases 77.5%, (93/120) were HIV+ (AKS) and 22.5% (27/120) HIV- (EKS) [χ^2 ^= 28.02, p < 0.001]. However, most sera from patients with reactive conditions (70.0%, 28/40) were also HIV+ but most (95.8%, 23/24) non-KS tumor patients were HIV negative. The difference between HIV infection amongst KS and non-KS tumor patients was statistically significant (two-tailed p-value = 0.046) [Table 1].

### HHV-8 serology

**Figure 2:**
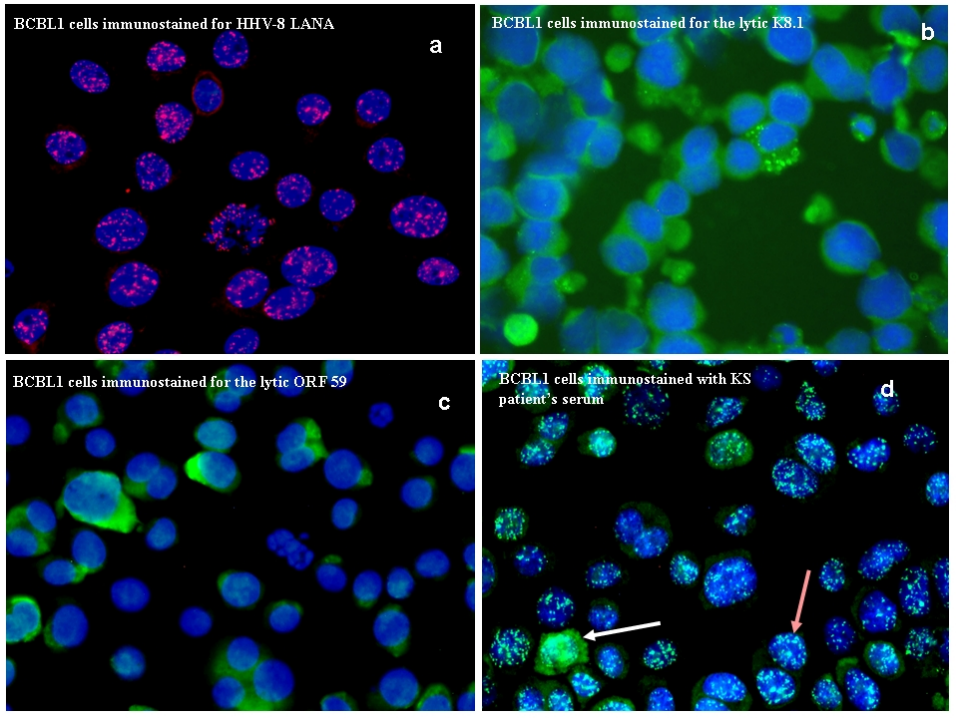
**IFA microphotographs showing BCBL-1 cytospin cells reactivity to anti-HHV-8 LANA (pink intranuclear dots) [a] and to lytic (green diffuse cytoplasmic staining) antibodies K8.1[b] and ORF 59 [c] 72 hrs after treatment with tetra-phobol acetate (TPA) to induce the lytic phase. KS patient's serum was added to BCBL-1 TPA treated cytospin cells producing, the lytic green diffuse cytoplasmic staining and latent white intranuclear dots. Note that majority of BCBL-1 cells reacted to latent (brown arrow) compared lytic (white arrow) antibodies in the index KS patient in [d], all sections × 400.**

The HHV-8 serology results are summarized in table 3 and 4. Most (89.0%, 164/184) screened cohort sera were HHV-8 seropositive based on either IFA (Figure 2) or ELISA indicating a high prevalence among this cohort of MNH patients [Table 3 and 4]. HHV-8 seroprevalence was highest (93.3%, n = 112/120) for KS cases, followed by non-KS tumors (91.7%, n = 22/24) and lowest (75.0%, n = 30/40) in non-malignant conditions. This difference in HHV-8 seroprevalence between the three disease groups was statistically highly significant (χ^2 ^= 8.35, p = 0.0039) [Table 3].

**Table 3:** Summary of HIV and HHV-8 screening among MNH patients by either IFA or ELISA between 1990 and 2001

	**Assay**	**IFA**		**ELISA**		
				
**Disease group**	**Serostatus**	**HHV8+**	**HHV8-**	**HHV8+**	**HHV8-**	**Total**
**KS**	**HIV+**	42 (45.2)	3 (3.2)	46 (49.5)	2 (2.2)	93 (77.5)
	**HIV-**	10 (37.0)	2 (7.4)	15 (55.6)	0	27 (22.5)
	**All cases**	**52 (43.3)**	**5 (4.2)**	**61 (50.8)**	**2 (1.7)**	**120 (100.0)**
**Non-KS tumors**	**HIV+**	1 (100.0)	0	0	0	1 (4.2)
	**HIV-**	8 (34.8)	1 (4.3)	13 (56.5)	1 (4.3)	23 (95.8)
	**All cases**	**9 (37.5)**	**1 (4.2)**	**13 (54.2)**	**1 (4.2)**	**24 (100.0)**
**Non-neoplastic**	**HIV+**	11 (39.3)	3 (10.7)	14 (50.0)	0	28 (70.0)
	**HIV-**	0	10 (83.3)	2 (16.7)	0	12 (30.0)
	**All cases**	**11 (27.5)**	**13 (32.5)**	**16 (40.0)**	**0**	**40 (100.0)**

	**Total**	**72 (39.1)**	**19 (10.3)**	**90 (49.0)**	**3 (1.6)**	**184 (100.0)**

**Table 4:** Association between HIV and combined (IFA and ELISA) HHV-8 sero-reactivity of studied patients at MNH (1990–2001)

		**HHV8 [no. (%)]**		**Total [no. (%)]**
			
	**Antibody Serostatus**	**Positive**	**Negative**	
		
**HIV**	**Positive**	114 (70.3)	8 (40.0)	**122 (66.3)**
	**Negative**	48 (29.7)	14 (80.0)	**62 (33.7)**
		
	**Total**	**162 (88.0)**	**22 (12.0)**	**184 (100.0)**

**Figure 3:**
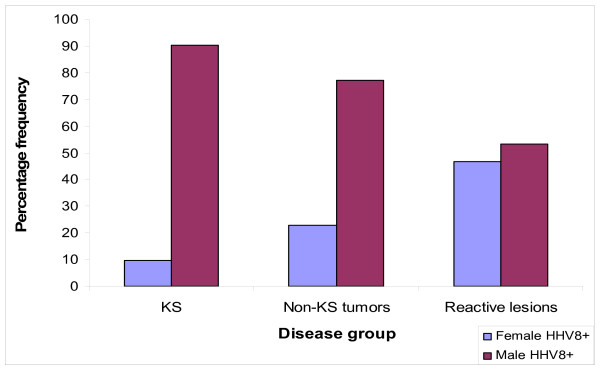
Total HHV-8 antibody seroprevalence by sex and disease groups among MNH patients (1990–2001).

Sex data was available for 154 HHV-8 seropositive cases and most (81.2%, 125/154) of them were males and only 18.8% (29/154) females. Male HHV-8 seroprevalence was particularly high (90.2%, 92/102) among KS patients and less among non-KS tumors (77.3%, 17/22) and non-neoplastic conditions (53.3%, 16/30) which difference appeared statistically highly significant (χ^2 ^= 20.86, p = 0.00003) [Figure 3].

**Figure 4:**
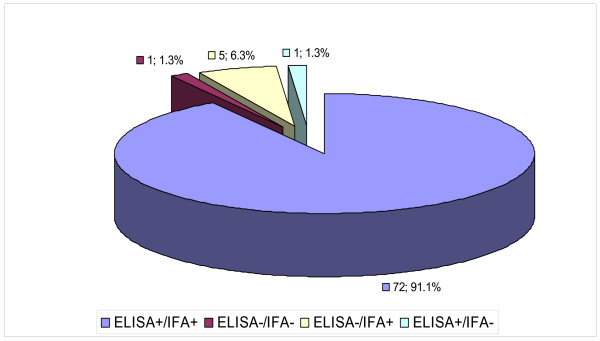
ELISA/IFA percentage sero-reactivity among MNH patients between 1990 and 2001.

Of all HHV-8 serology tests, 79 specimens were assayed by both IFA and ELISA and 92.4% (73/79) showed concordance and most (91.1%, 72/79) were ELISA+/IFA+ as well as one case (1.3%, 1/79) double negative [Figure 4]. However, 7.6% (6/79) the specimens were ELISA/IFA discordant including those ELISA-/IFA+ (6.3%, 5/79), and one ELISA+/IFA- case (1.3%, 1/79) [Figure 4]. Thus, although IFA appeared to have a higher (98.6%) assay sensitivity [confidence interval (CI) = 91.6–99.9%], it also showed lower specificity (16.7%, CI = 0.9–63.5) and positive predictive value (PPV) [93.5%, CI = 84.8–97.6], compared to ELISA which showed a reasonably high (93.5%, CI = 84.8–97.6) sensitivity, as well as higher PPV (98.6%, CI = 91.6–99.9) and specificity (50.0%, CI = 2.7–97.3). However, it appears [Figure 2(a–d)] that IFA can clearly visualize latent [latency-associated nuclear antigen (LANA)] [Figure (2a &2d)] as well as lytic [Figure 2b &2c] antibody reactivity allowing quantification of both latent and lytic HHV-8 infection within one individual [Figure 2(d)].

### HIV and HHV-8 correlations

Evidently, most (70.3%, 114/162) HHV-8 seropositive patients appeared also, co-infected with HIV and conversely, most (80.0%, 12/20) of those HHV-8 seronegative were also non-reactive for anti-HIV antibodies which difference appeared statistically highly significant (P = 0.003 Fisher Exact Test) [Table 4].

### Immunohistochemistry (IHC)

**Figure 5:**
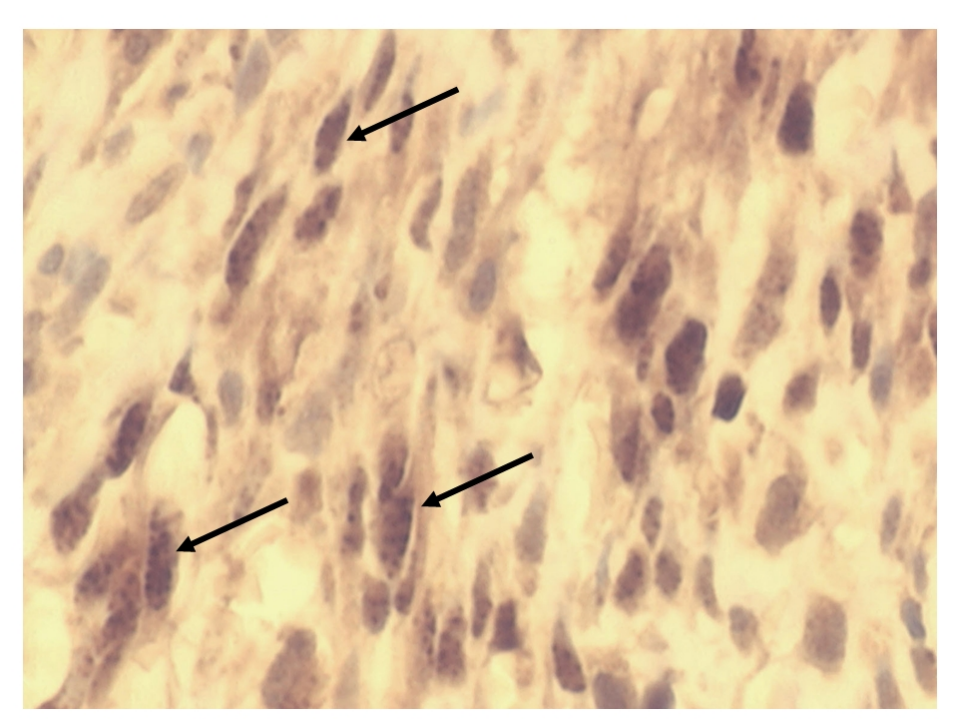
A section of an immunoperoxidase stained nodular cutaneous AKS lesion showing HHV-8 LANA+ granular nuclear reactivity in SC (black arrows) × 600.

All early stage KS including patch (17.5%, 21/120) and plaque (14.2%, 17/120) were cutaneous and HHV-8 LANA+ (Figure 5) whereas two apparently HHV-8 seronegative KS were nodular stage. Similarly, although all the HHV-8 positive KS sera came from patients with LANA+ biopsies (Figure 5), about 6.7% (8/120) of corresponding KS sera were HHV-8 negative indicating a tissue-serum discrepancy.

## Discussion

All the three studied Tanzanian disease groups showed comparable demographical features and the male predominance in all KS types is concordant with previous findings by us and others [5,6,8]. The relatively high seroprevalence for HHV-8/KSHV in patients with non-KS tumors and non-neoplastic (reactive) conditions has not been previously documented in Tanzania and is compatible with a relatively high endemic incidence of HHV-8 infection [5,6].

The rather high prevalence of HIV infection in two-thirds of the studied cohort patients at MNH is alarming but also reflects a KS diagnostic selection bias. The dramatic (8-fold) difference in the male:female ratio of EKS (1:27) and AKS (1:3) confirms previous reports from Tanzania, eastern Africa and elsewhere [8,11-15]. This sex ratio decline in AKS apparently, reflects an increased frequency of HIV infected sexually active females, compared to males of the same age group and a suggested biological gender resistance for females to non-HIV forms of KS [EKS, classic (CKS) and iatrogenic (IKS)] which clearly show a male predominance particularly in homosexuals [2,8,11,14,16,17]. The finding that most non-KS tumors in our selected cohort were HIV negative emphasizes the notion that essentially, KS today is an AIDS-defining malignancy.

As expected by their KS diagnosis, the HHV-8 seroprevalence (90%) in the studied cohort sera was higher than that in the healthy non-hospitalized population of about 50% in Tanzania [5,6] and of 70% in sub-Saharan Africa [4]. The slightly lower seroprevalence observed in our previous PCR study could be attributed to the small KS sample size compared to that in our present study [6] but differences in assay sensitivity/specificity should also be considered [4]. Obviously, this high HHV-8 frequency in patients at MNH implies a potential high risk of parenteral/iatrogenic transmission for both patients and staff, particularly during blood transfusion and other invasive procedures, as well as in intravenous (IV) drug users [4,5,18].

The higher (93%) HHV-8 prevalence among KS cases compared to non-KS tumors and reactive lesions supports a causal relationship between the virus and primarily KS but other lymphoid tumors as primary effusion lymphoma (PEL) or body cavity-based lymphoma (BCBL) and multicentric Castleman's disease (MCD) which may also be associated with HHV-8 [2,5,19] were surprisingly not evident in our study.

Evidently, the finding that HHV-8 was more prevalent in males in all disease groups is concordant to our previous reports and others [5,6,8,11,20] on HHV-8 seroprevalence in KS and that KS is predominantly a male disease in cutaneous AIDS-associated (AKS), African endemic (EKS), classical (CKS) and iatrogenic [IKS]/post-transplantation Kaposi's sarcoma [6,8,11,20] although, we have recently reported an increased frequency of females with oral AKS (OAKS) [8]. Furthermore, we have shown previously that HHV-8 is not only more frequent in males, but that they appear to have higher viral (HHV-8) loads than females in both sera and tissue lesions by both immuhistochemistry and real-time PCR [6,8]. Reasons for this sex differences are yet not fully clarified although various biological and socio-behavioral factors were suggested [6,8]. Recently in a comparative genomic hybridization (CGH) and interphase-fluorescence *in-situ *hybridization (interphase FISH) study, we have reported cytogenetic differences between male and female KS patients including the loss of chromosome Y observed in all early and the majority of late male AKS and EKS representing a clonal genomic change [21]. Moreover, in early stage disease loss of Y chromosome was the only recurrent change found [21]. These features are apparently, mainly related to early male KS pathogenesis and may therefore indicate cytogenetic reasons for the sex differences in KS [21]. Furthermore, the higher seroprevalence of the oncogenic HHV-8 among male KS patients may also relate to the higher frequency of KS in males [6,8,20,21].

Interestingly, the male predominance for HHV-8 seroprevalence in our current study was also seen in non-KS and non-neoplastic patients suggesting a possible higher susceptibility/permissiveness of males to KSHV/HHV-8 infection and/or a high viral transmission among male homosexuals and intravenous (IV) drug users usually, mostly males [8,18,22,23]. However, the association of sex with KS development and HHV-8 infection is yet to be fully elucidated.

Our finding that HHV-8 antibody seroprevalent patients at MNH were 3-times more often HIV co-prevalent than the HHV-8 seronegative cases is consistent with other reports [4,18,24] indicating corresponding seroepidemiology for these viruses, partly explaining the much increased risk of KS development among those HIV and HHV-8 co-infected as compared to those infected with either or neither of the viruses [2,4,8,18,24]. Furthermore, the findings support also the notion of HIV and HHV-8 cross-talk, partly achieved through the transactivation of KSHV by HIV-Tat as reported previously by us and others [2,6,9,25]. This HIV-HHV8 cross-talk, calls for exploration for the development and application of possible combined anti-HIV, anti-HHV-8 and anti-KS prevention and therapeutic strategies among high-risk groups including HIV-infected and AIDS patients.

The finding that IFA showed greater sensitivity than ELISA is probably due to that IFA methods shows antibodies to both lytic and latent antigens and that lytic are reportedly more sensitive [5]. However, our finding that ELISA had apparently, higher positive predictive value as well as specificity and still had a high sensitivity was unexpected and particularly favourable as it makes HHV-8 screening more affordable in a resource-constrained country like Tanzania, mostly lacking the cell culture and fluorescence microscopy facilities needed in IFA. Consequently, ELISA can allow larger-scale screening of HIV high-risk groups including blood/organ donors and thereby prevent KS development through early antiviral interventions (including vaccination) [4,5]. However, these findings are prone to observer errors, sampling bias (hospital data) including the fact that HHV-8 screening is a field that is still evolving methodologically [4]. Nevertheless, the IFA method also appears to afford visual evaluation of the percentage seroprevalence of lytic as well as latent anti-HHV-8 antibodies apparently, allowing categorization of patients as being in the productive and potentially infective (lytic) and non-productive but oncogenically more significant latent phase [2] and may therefore be useful when such characterization is desired for clinical or public health interventions.

Our finding that all sera from early stage (patch-plaque) KS were positive for anti-HHV-8 antibodies while all negative sera were from LANA+ biopsies of nodular KS patients, further supports our previous notion of stage-dependent tissue-serum discrepancy in viral antigen and antibody expression probably due to virus tissue retention, immune-segregation and/or selective clearance during KS evolution [6,19].

Again, the clear relationship between HHV-8 and HIV infections and KS prevalence underlines the interaction between the two viruses being possibly facilitated in part, by similar transmission patterns and also, their transactivation capacity [2,4,6,19].

## Conclusion

HHV-8 seroprevalence in patients at MNH Tanzania appears to be high as expected, in HIV+ and HIV- KS cases and males but also in the HIV seronegative non-KS tumors. ELISA showed a combination of high HHV-8 sensitivity as well as higher PPV and specificity compared to IFA which however showed higher sensitivity. An apparent stage-dependent tissue-serum discrepancy in HHV-8 antigen and antibody expression seems to support the notion of immune-segregation and/or selective virus clearance during the evolution KS. HHV-8 screening of patients at risk of KS and of blood and organ donors particularly in high endemic areas will evidently help prevent development of KS.

## Methods

### Biopsies

Diagnostic archival biopsies fixed in 10% neutral buffered formalin and paraffin-embedded (FFPE) and corresponding, available sera and medical records of KS patients between 1990 and 2001 were retrieved and evaluated [Dept. Histopathology, Muhimbili National Hospital (MNH)/Muhimbili University of Health and Allied Sciences (MUHAS), Dar es Salaam] and at the Immunopathology Lab [Karolinska University Hospital Solna, Stockholm]. Furthermore, available biopsies and corresponding sera from non-KS tumors and reactive conditions from the same period were also evaluated. The biopsies of reactive conditions included all histologically non-malignant/inflammatory cases whose sera were available for analysis. None of the patients had received any antiretroviral (ARV) or anti-tumour therapy before the biopsy was taken.

### Histology

Routine hematoxylin and eosin (H & E) staining was done as previously described [8,26] and microscopic evaluation was done independently by three pathologists as previously described [8].

### Serology

Available sera from patients with histologically diagnosed KS, non-KS malignancies as well as reactive conditions were comparatively evaluated for HIV and HHV-8 serology. Serology for HIV-1 evaluation (ELISA) was performed at Microbiology/Immunology MUHAS as previously described [8,27,28].

HHV-8 serology by IFA was performed [Swedish Institute for Infectious Diseases Control (SMI)] on cytospins of BCBL-1* cells [29] using patients sera as well as control lytic (K8.1 and ORF 59) and latent (ORF 73 or LANA) antibodies [Advanced Biotechnologies Inc. (Columbia, MD)] as previously described [5,8]. Results were evaluated and documented by microphotography (Immunopathology Lab).

The HHV-8 infected body cavity-based lymphoma (BCBL-1) cells (kindly provided by G. Gaidano) were derived from a primary effusion lymphoma (PEL) [30,31], and cultured [Immune and Gene Therapy Lab., Cancer Center Karolinska (CCK)] in RPMI 1640 medium (Gibco, BRL, UK) containing 10% heat-inactivated FCS serum, 2 mM L-glutamine, 100 IU/ml penicillin and 100 μg/ml streptomycin at 37°C and 5% CO_2_. To induce lytic gene transcription, cells were cultured with 20 ng/ml of 12-*O*-tetradecanoylphorbol-13-acetate (TPA) [Sigma Chemical Co., St. Louis, Mo.] as previously described, [5,28,32] and harvested after 72 hours, washed with PBS and fastened by cytospin on SuperFrost^® ^slides (Menzel GmbH & Co KG, Braunschweigh, Germany). The slides were fixed for 10 minutes in 4% paraformaldehyde (PFA) and washed before immunostaining as previously described [5,8].

For HHV-8 antibody evaluation an IgG enzyme immunoassay (EIA) Kit (96 Wells, HHV-317-02) Biotrin International (Dublin, Ireland) was used. The Biotrin HHV-8 ELISA is a direct EIA based on the binding of HHV-8-specific antibodies to lytic peptide antigens coupled to microtitre test strips. Bound antibodies are detected by an anti-human IgG peroxidase conjugate and a 3,3',5,5'-tetramethylbenzidine (TMB) dark blue substrate reaction (BioFX Laboratories, Inc. Owings Mills, MD). The use of lytic peptide epitopes derived from various HHV-8 viral proteins ensures both a high sensitivity and specificity. The Kit has no detectable cross-reactivity with HIV/EBV antibodies. Control sera included those from known HHV-8+ KS patients while the HHV-8 infected BCBL-1 cells provided an internal positive control for IFA studies. The negative controls for both IFA and ELISA included sera from known HIV and HHV-8 negative non-KS patients and a buffer (PBS).

### Immunohistochemistry (IHC)

Immunostaining with the avidin-biotin complex (ABC) immunoperoxidase technique was used on KS sections (5 μm) for detection of HHV-8 latency-associated nuclear antigen (LANA) to compliment histopathology and serology, as previously described [8,19,26,33]. Negative controls included sections from non-KS tissues as well as incubation with PBS instead of the primary antibody. Positive controls included previously tested KS sections and FFPE sections of HHV-8 LANA+ cells from a body cavity-based lymphoma (BCBL-1). The number of LANA+ cells/HPF (× 400 magnification) was evaluated on micrographs as previously described [8,19,33].

### Microscopy

For microscopy and microphotography an Olympus BX60, microscope with a digital camera (Sony DKC-5000) was used as previously described [8,19,33].

### Statistical Analysis

The EPI INFO 6 statistical software programme (CDC, Atlanta, GA) was used. P-values of ≤0.05 were considered statistically significant. The Fisher exact test was used to test the significance level where the sample size was small.

## Competing interests

The authors declare that they have no competing interests.
